# Supplementary material for: Complexome profiling of the Chlamydomonas psb28 mutant reveals TEF5 as an early PSII assembly factor
Source: Plant Cell. 2025 Mar 17;37(6):koaf055. doi: 10.1093/plcell/koaf055 (PMC12164590; doi:10.1093/plcell/koaf055)
Supplement: koaf055_Supplementary_Data [file koaf055_supplementary_data.zip › psb28_interactive.zip!/plots/intensity/qualityPlots/peptides/_Cre02.g099601.t1.1.html]

### Cre02.g099601.t1.1

Trivial name:   
SeqCoverage [median]: 0.80  
Mass: 351.33 kDa  
  
 

| Sequence | MissedCleavage | Mass [Da] | wtIntensity | muIntensity | Score |
| --- | --- | --- | --- | --- | --- |
| IEAVPADAAR | 0 | 1011.53 | 955.32 | 5914.17 | 71.34 |
| LPAGGGVR | 0 | 725.42 | 676.92 | 0.00 | 94.67 |
|  |  |  | 1632.2 | 5914.2 |  |
